# Characterisation of urokinase plasminogen activator receptor variants in human airway and peripheral cells

**DOI:** 10.1186/1471-2199-10-75

**Published:** 2009-07-28

**Authors:** Ceri E Stewart, Ian Sayers

**Affiliations:** 1 Division of Therapeutics and Molecular Medicine, Nottingham Respiratory Biomedical Research Unit, University of Nottingham, Queen's Medical Centre, Nottingham, NG7 2UH, UK

## Abstract

**Background:**

Expression of the urokinase plasminogen activator receptor (*UPAR*) has been shown to have clinical relevance in various cancers. We have recently identified *UPAR *as an asthma susceptibility gene and there is evidence to suggest that uPAR may be upregulated in lung diseases such as COPD and asthma. uPAR is a key receptor involved in the formation of the serine protease plasmin by interacting with uPA and has been implicated in many physiological processes including proliferation and migration. The current aim was to determine key regulatory regions and splice variants of *UPAR *and quantify its expression in primary human tissues and cells (including lung, bronchial epithelium (HBEC), airway smooth muscle (HASM) and peripheral cells).

**Results:**

Using Rapid Amplification of cDNA Ends (RACE) a conserved transcription start site (-42 to -77 relative to ATG) was identified and multiple transcription factor binding sites predicted. Seven major splice variants were identified (>5% total expression), including multiple exon deletions and an alternative exon 7b (encoding a truncated, soluble, 229aa protein). Variants were differentially expressed, with a high proportion of E7b usage in lung tissue and structural cells (55–87% of transcripts), whereas classical exon 7 (encoding the GPI-linked protein) was preferentially expressed in peripheral cells (~80% of transcripts), often with exon 6 or 5+6 deletions. Real-time PCR confirmed expression of uPAR mRNA in lung, as well as airway and peripheral cell types with ~50–100 fold greater expression in peripheral cells versus airway cells and confirmed RACE data. Protein analysis confirmed expression of multiple different forms of uPAR in the same cells as well as expression of soluble uPAR in cell supernatants. The pattern of expression did not directly reflect that seen at the mRNA level, indicating that post-translational mechanisms of regulation may also play an important role.

**Conclusion:**

We have identified multiple uPAR isoforms in the lung and immune cells and shown that expression is cell specific. These data provide a novel mechanism for uPAR regulation, as different exon splicing may determine uPAR function *e.g*. alternative E7b results in a soluble isoform due to the loss of the GPI anchor and exon deletions may affect uPA (ligand) and/or integrin binding and therefore influence downstream pathways. Expression of different isoforms within the lung should be taken into consideration in studies of uPAR in respiratory disease.

## Background

The plasminogen pathway and specifically the urokinase plasminogen activator receptor (uPAR) has been of interest in the cancer field for some time (reviewed in [1,2]). The uPAR protein has been shown to be over-expressed in many different cancers [2], whilst an exon 4–5 deleted mRNA has been shown to be over-expressed in breast cancer [3].

The uPAR protein is a modulator of the plasminogen pathway, which cleaves and activates urokinase (uPA) [4]. uPAR-uPA is involved in the proteolytic activation of plasminogen to plasmin, which in turn degrades fibrin and other ECM components and activates matrix metalloproteases. However uPAR has many other roles (reviewed in [5,6]), acting via both intra- and extra- cellular signalling pathways. For instance, soluble uPAR fragments have chemotactic activity [7], whilst uPAR interacts with integrins [8] and G-protein coupled receptors to signal within the cell via intracellular kinases. These many interactions explain the position of uPAR as a key player in tissue remodelling, proliferation and cell migration, in both normal and disease states [5].

A key feature of many respiratory diseases, including asthma, is airway remodelling [9]. From the known biology of uPAR, this receptor is potentially involved in tissue remodelling and may be predicted to influence decline in lung function, due to its role in ECM remodelling, proliferation and migration of several cell types [10]. Recently we have performed linkage and association analyses utilising three populations including 587 UK and Dutch asthma families and identified the urokinase plasminogen activator receptor gene (*uPAR *or *PLAUR*) as an asthma susceptibility gene [11]. We have shown that single nucleotide polymorphisms (SNPs) spanning *UPAR *determine the rate of decline in FEV1 and are also determinants of soluble uPAR in the serum of asthma subjects [11]. In addition, uPAR has been shown to be up-regulated in lung epithelium in severe/fatal asthma [12], whilst soluble uPAR was found to be elevated in the sputum in multiple respiratory diseases (asthma, chronic obstructive pulmonary disease (COPD) and cystic fibrosis) [13]. A gene profiling study also showed uPAR expression was related to reduced lung function in COPD patients, with increased staining for the uPAR protein in the airway epithelium of airflow obstructed patients [14]. Therefore our previous data and data from others suggest that levels of uPAR expression may be important in multiple respiratory diseases.

**Figure 1:**
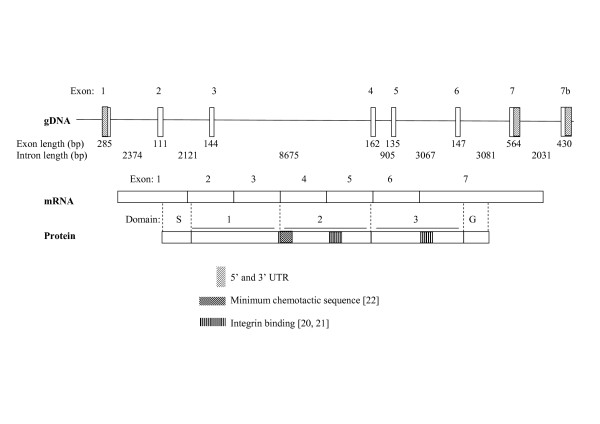
**Schematic representation of uPAR structure**. *UPAR *consists of seven exons, with an alternative exon 7b previously reported [24]. The classical form is transcribed to give a three domain protein which can be membrane bound, via a GPI anchor. Domain S: Signal peptide removed during processing, Domain G: removed during processing to give GPI anchor at new *C-*terminus. Different regions of each domain involved in various protein-protein interactions are highlighted, which may give an indication of the functions of variant forms of the receptor. uPA binding involves residues in all three domains (see Figure 4), with key binding regions in D1 and D2 [17,19]. The minimum chemotactic sequence is located in the D1-D2 linker [22], A region implicated in binding integrins α1β3 and α5β1, aiding signalling to vitronectin, which also has chemotactic activity is located in D2 [20]. A region in D3 which binds integrin α5β1 is also highlighted [21].

The uPAR protein has three domains and is bound to the membrane via a glycosylphosphatidylinositol (GPI) anchor; however it may also be found as a soluble form due to cleavage of the GPI anchor and both membrane bound and soluble forms may be cleaved by various proteases, including uPA to give D1 and D2/3 fragments [15]. The human airway trypsin-like protease (HAT) expressed in airway epithelium can cleave both membrane bound and soluble uPAR at the D1/D2 linker [16], resulting in release of the D1 fragment. The mature uPAR protein consists of three similar domains which assemble to create a globular structure almost surrounding a central cavity in which uPA binds [17,18]. Specific residues have been determined to be involved in binding uPA [17,19] and integrins [20,21] as well as having chemotactic functions [22] (Figure 1).

The *UPAR *gene consists of seven exons. Exon 1 encodes the 5'UTR and a signal peptide, whilst exons 2–3, 4–5 and 6–7 encode the homologous protein domains D1, D2 and D3 [23]. Use of an alternative exon 7 (described hereafter as exon 7b) has been reported and results in replacement of the terminal 75 amino acids with a novel 21 residues; this variant cannot gain a GPI anchor and thus encodes a soluble form of the receptor [24]. Variants lacking exons 5 and 4+5 have also been described [3,22,23]. The exon 5 variant was originally identified from a HeLa cDNA library whilst the exon 4+5 deletion was identified in various cancer cell lines using a limited PCR design. To date no comprehensive evaluation of splice variation of uPAR has been completed.

Due to the increasing interest in uPAR expression in lung diseases, the current study aimed to characterise the *UPAR *gene and identify cell specific transcription initiation sites as well as to identify and quantify splice isoforms in cells and tissues from the lung and periphery. These data provide definitive answers regarding the type and extent of uPAR splice variation and generate a greater insight into the molecular mechanisms underlying the expression and activity of uPAR in the normal lung and immune cells, which may in turn have implications for the role of this receptor in airway disease.

## Results

### *UPAR* has a localised transcriptional start site that shows limited cell specificity

**Figure 2:**
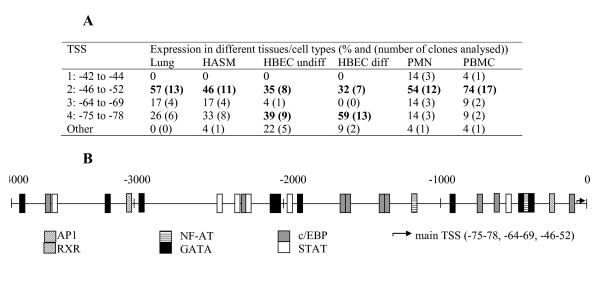
**Identification and characterisation of the uPAR promoter**. (A) 5' RACE performed on a panel of tissues/cell types identified a cluster of transcriptional start sites (TSS) which show distinct patterns of usage. TSS are listed relative to ATG of translated sequence. (B) 4000 bp upstream (from ATG) was analysed for potential transcription factor binding sites using four different programs. Those sites identified in two or more different searches are shown.

The transcriptional start sites (TSS) for *UPAR *were determined by 5' RACE in a panel of six human tissues/cell types (Figure 2A). In lung tissue, human airway smooth muscle cells (HASM), polymorphonuclear cells (PMN) and peripheral blood mononuclear cells (PBMC), the dominant TSS was located at -46 to -52 bp relative to the start codon, however the major TSS in differentiated human bronchial epithelial cells (HBEC) was at -75 bp and a further two minor sites were identified at -42 to -44 (in PMN/PBMC) and at -64 to -69 (all except differentiated HBEC). Differences in TSS usage between tissues/cells were significant (Chi-square, p < 0.001). A shortlist of transcription factors relevant to respiratory disease and asthma was generated by reference to the literature and a 4 kb upstream fragment analysed for potential transcription factor binding sites using four different on-line databases. Consensus sites for c/EBP (9), AP-1 (1), GATA (9), NF-AT (2), STAT (6) and RXR (1) were identified (Figure 2B).

### uPAR is expressed in multiple splice forms in the lung and periphery

**Figure 3:**
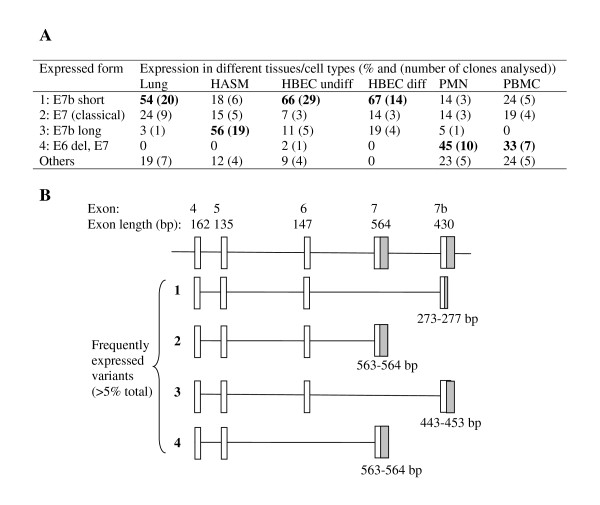
**Identification of 3' variants in a panel of tissues/cell types**. 3' RACE was performed on a panel of tissues/cell types using a uPAR-specific forward primer located in exon 4. Frequencies of the most common variants (>5% overall expression) in each cell type based on numbers of RACE clones are summarised (A) and their structures shown (B). "Other" variants include both alternative termination sites and splice variants. Grey blocks highlight 3' UTR in the terminal exon.

**Figure 4:**
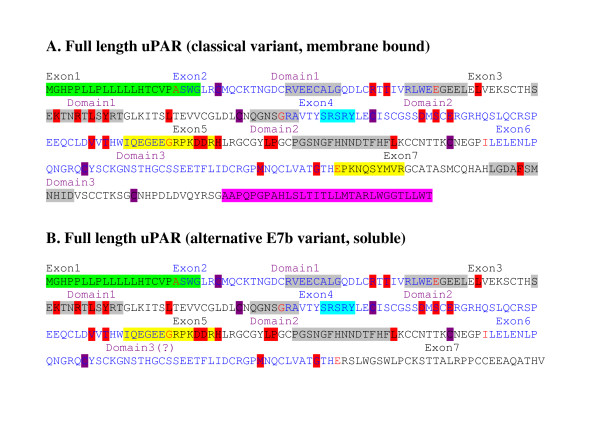
**Protein sequences of uPAR exon 7 splice variants**. Alternate splice variants which were identified and confirmed by real time PCR in the current analyses are shown. Alternate exons are labelled back and blue; red amino acids are encoded over an exon boundary. The signal peptide, removed during processing (not included in numbering of mature peptide) is highlighted green. The region highlighted pink is removed during processing to give GPI anchor at the new *C-*terminus. Domains are defined by the end cysteines involved in disulphide bridges (purple). Peptide D2A (yellow, domain 2) binds integrins αvβ3 and α5β1, aiding signalling to vitronectin and also has chemotactic activity [20], whilst a region highlighted in domain 3 (yellow) binds integrin α5β1 [21]. The minimum chemotactic domain (turquoise) binds FPRL1 and encourages chemotaxis of many cell types [22]. uPA binding regions determined by phage display and peptide array are highlighted grey [19], whilst residues involved in the uPA binding determined by alanine scanning mutagenesis are highlighted red [17].

**Figure 5:**
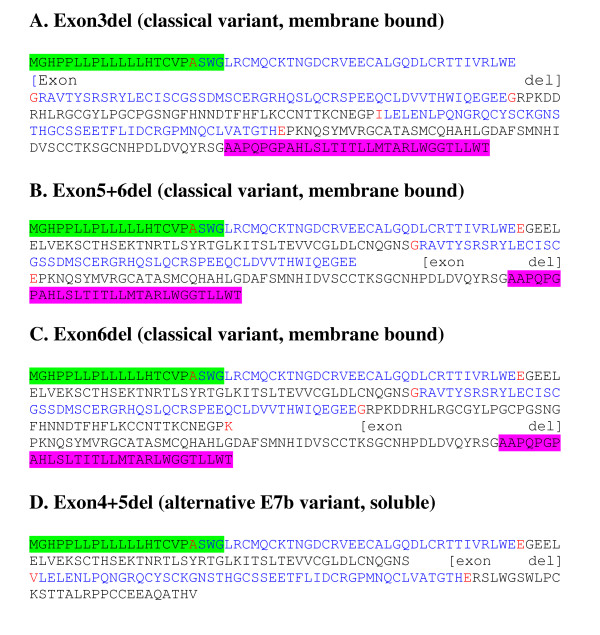
**Protein sequences of uPAR internal exon splice variants**. Alternate splice variants which were identified and confirmed by real time PCR in the current analyses are shown. A signal peptide removed during processing (not included in the numbering of mature peptide) is highlighted green and the terminal domain (pink) is removed during processing to give a GPI anchor at the new *C-*terminus.

To identify splice variants and determine the exon structure of uPAR, 5' and 3' RACE were completed in six different tissues/cell types (Figures 2 and 3). 5' RACE identified a localised TSS (see earlier) and demonstrated that exons 1 and 2 were conserved in all cells/tissues (data not shown). An exon 3 deletion variant was identified at low frequency in differentiated HBEC (4.5% of clones sequenced), PMN (4.5%) and PBMC (4.3%). 3' RACE confirmed the expression of the classical exon 7 as well as the alternative exon 7b, which was present with a truncated or extended 3'UTR (Figure 3). The pattern of expression of splice variants differed between tissues/cell types (Chi-square p < 0.001). Several exon deletion variants were identified including; exon 5, 6 and 5+6 deletions, although only the exon 6 deletion had a frequency >5% (Figure 3). In addition PCR products spanning exons 1–7 and 1–7b were cloned and sequenced to determine which exon deletions were found in combination with the two alternative terminal exons, demonstrating that an additional exon 4+5 deletion exists (data not shown). Interestingly, the structural cell types (HASM, HBEC) and lung appeared to express mostly exon 7b (55–87% of clones sequenced), encoding a soluble variant of uPAR, whilst more classical membrane bound uPAR (≈ 80%) was detected in the peripheral cells (PMN and PBMC) (when all exon deleted forms were pooled). In RACE, exon-deleted transcripts were cloned from all cell types except differentiated HBEC and HASM. These results were confirmed by full-length PCR, when no exon-deleted forms were obtained for differentiated HBEC and 2/48 clones for HASM, which would not have been detectable by RACE based on primer design (del3 and del4+5). Lung tissue showed 8% exon deleted clones in the RACE analysis; these were exon 5 or 6 deletions, in combination with exon 7b. Of the lung cells, undifferentiated HBEC showed the largest number of exon-deleted forms (7/44 clones analysed, 16%), which included three unique clones showing non-classical splice sites (data not shown). The two peripheral cell types analysed showed high frequencies of exon deleted forms in the RACE analysis (PMN: 14/22, 64%; PBMC: 10/21, 48%). These were predominantly exon 6 deletions, although del5 and del5+6 were also observed. PCR analysis of PBMC confirmed these data with 16/24 exon1–7 clones showing exon deletions (including del6 (5/24), del5 (4/34) and del5+6 (3/24)) and 4/24 exon 1–7b clones showing deletions (including del6 (1/24) and del5 (2/24)). All classical exon deletions are in frame deletions. See Table 1 for molecular details of splice variation and Figures 4 and 5 for predicted protein sequences.

**Table 1:** Alternative uPAR splicing identified in the RACE analyses

Variant	5'Donor site	3'Acceptor site	Implication
E3 deletion	GCGCTTGTGGGAAG	GCCGGGCTGTCAC	In frame deletion, removal of 48 residues
E4+5 deletion	CCAGGGCAACTCTG	TCCTGGAGCTTGA	In frame deletion, removal of 100 residues, valine insertion
E5 deletion	AGGTGAAGAAG	TCCTGGAGCTTGA	In frame deletion, removal of 46 residues, valine insertion
E6 deletion (with E7)	CAACGAGGGCCCAA	AACCGAAAAACC	In frame deletion, removal of 50 residues, lysine insertion
E6 deletion (with E7b)	CAACGAGGGCCCAA	AACGCTCACTCTGG	In frame deletion, removal of 50 residues, lysine insertion
E5+6 deletion (with E7)	AGGTGAAGAAG	AACCGAAAAACC	In frame deletion, removal of 94 residues
E3+4+5 deletion	GCGCTTGTGGGAAG	TCCTGGAGCTTGA	In frame deletion, removal of 148 residues, valine insertion

uPAR variants detected at >5% frequency in RACE analysis are included.

### Real-time quantitative PCR confirms uPAR splice variants and identifies cell-specific expression

**Figure 6:**
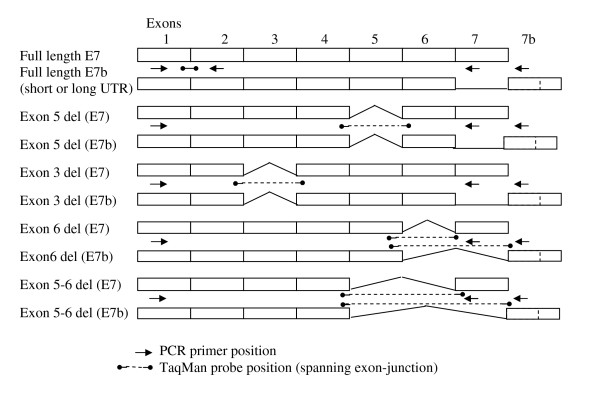
**Location of Taqman primers and probes**. Variants shown were identified in one or more clone and in one or more cell type during RACE/PCR analyses. Location of primers and probes generating variant specific TaqMan assays are indicated.

In order to confirm and quantify the expression of different uPAR variants at the mRNA level, a series of real-time PCR assays were designed to amplify uPAR exons 1–2 (conserved, total uPAR), 1–7 and 1–7b, whilst a series of deletion-specific probes allowed detection of variants (Table 2 and Figure 6). Two different normalising or housekeeping assays, 18s ribosomal RNA and hypoxanthine guanine phosphoribosyltransferase 1 (HPRT1), were tested and showed similar results (data not shown). However, HPRT1 was used in the main analysis as its level of mRNA expression was more comparable to that of uPAR itself.

**Table 2:** Primers and probes for RACE and real-time PCR

Primers			
Assay	Primer	Position	Sequence
RACE	5'RACE nested	48852556	CCTCTCACAGCTCATGTCTGAT
RACE	5'RACE	48852500	ACCACATCCAGGCACTGTTCTTC
RACE	3'RACE	48852519	GAACAGTGCCTGGATGTGGTGAC
PCR/Real-time	uPAR Fwd E1	48866085	CTGCTGCTGCTCCACACCT
PCR/Real-time	uPAR Rev E7	48845002	TGGGTGGTTACAGCCACTTT
PCR/Real-time	uPAR Rev E7b	48842344	ATCACATCTGCTGGGGCTAC
PCR/Real-time	uPAR Rev E2	48863624	ACTCTTCCACACGGCAATCCC
PCR/Real-time	uPAR Rev E7b (long UTR)	48842223	GCCTGTCCTTCCATGATGTT

Probes			
Assay	TaqMan Probe	Location	Sequence ('denotes exon boundary)

Real-time PCR	Exon 1–2	Exon1–2	CCAGCCTCTTGGGGCCTGCGGT
Real-time PCR	Exon 5 del	Exon4–6	AGCTCCAGGA'CTTCTTCACCTTCC
Real-time PCR	Exon 3 del	Exon 2–4	CAGCCCGGC'CTTCCCACAAGCG
Real-time PCR	Exon 6 del +E7	Exon 5–7	TTCGGTT'TTGGGCCCTCGTTGCAT
Real-time PCR	Exon 6 del +E7b	Exon 5–7b	TTCCCCAGAGTGAGCGTT'TTGGGC
Real-time PCR	Exon 5+6 del +E7	Exon 4–7	TCCAGGAAGGTGAAGAAG'AACCGAAA
Real-time PCR	Exon 5+6 del; E7b	Exon 4–7b	CCCAGAGTGAGCGTT'CTTCTTCACC
Real-time PCR	Exon 4+5 del	Exon 3–6	TGCAACCAGGGCAACTCTG'TCCT

Primers listed for RACE were used with RACE-specific primers from GeneRacer kit (Invitrogen). Other primers were used to amplify full-length uPAR for both standard PCR and real-time assays. All TaqMan probes were labelled with Fam and TAMRA and were used with appropriate primers. Primer location is listed relative to chromosome 19 reference sequence for the start of the primer.

**Figure 7:**
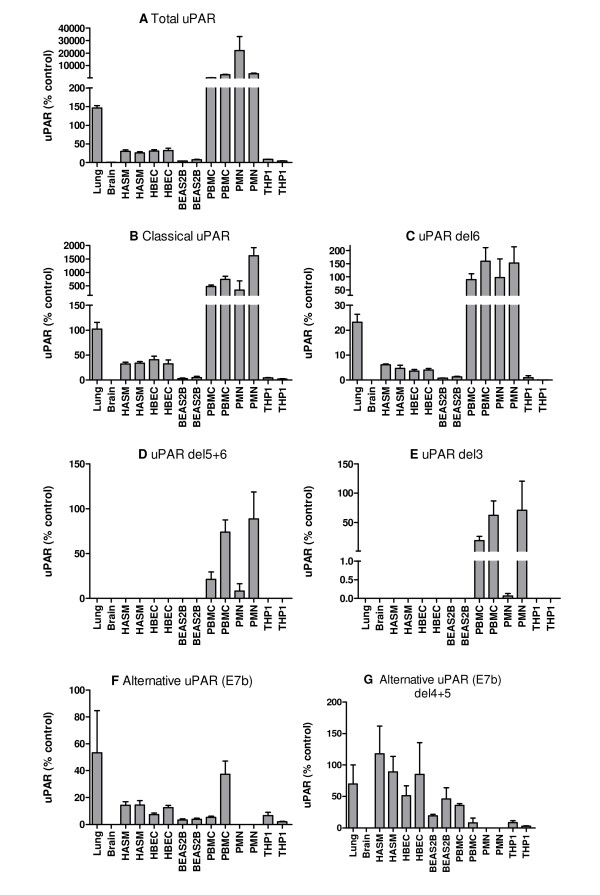
**Expression of uPAR splice variant mRNA in different tissues/cell types**. A series of real-time PCR (TaqMan) assays was used to measure the expression of different splice variants of uPAR in an extended panel of tissues/cell types (lung, brain, HASM, undifferentiated HBEC, BEAS2B, PMN, PBMC, THP1). Expression of each variant is shown as mean + SEM of three PCR replicates, for two donors or biological replicates as appropriate. Data are shown as 2^-ΔCt ^normalised to HPRT1 and relative to a suitable plasmid positive control containing the specific splice variant cDNA (designated 100%). (A) total uPAR, (B) total classical uPAR (exon 7), (C) classical uPAR exon 6 deletion, (D) classical uPAR exons 5+6 deletion, (E) classical uPAR exon 3 deletion, (F) total alternative uPAR (exon7b), (G) alternative uPAR exon 4+5 deletion. Classical uPAR exon 5 and 4+5 deletions and alternative uPAR exon 5 deletion were not detected.

Real-time PCR was performed using cDNA from eight tissues/cell types in total, from either one (lung and brain) or two (HASM, HBEC, PMN, PBMC) donors, or prepared from RNA extracted on two different occasions for cell lines (THP1 (monocyte), BEAS2B (bronchial epithelium)). Total uPAR expression was confirmed in the lung tissue and varied in a cell-type specific pattern; PMN ≥ PBMC>>lung>HBEC≈HASM>BEAS2B≈THP-1 (ANOVA p = 0.013), the HASM and HBEC expression levels being ~100 fold lower than primary peripheral cells (PMN and PBMC) (Figure 7A). Expression was very low in brain (6-fold less than cultured cells).

Overall, expression levels of classical uPAR (exon 7) correlate with total uPAR levels, PMN ≥ PBMC>>lung>HBEC≈HASM>BEAS2B≈THP1 (Pearson r = 0.891, p = 0.003), suggesting that this may be the dominant expressed variant (Figure 7A and 7B). Exon deleted versions of classical uPAR were also confirmed (Figure 7C – E). Exon 6 deletions were widely expressed with an expression profile similar to classical uPAR (exon 7) expression, whilst exon 3 and 5+6 deletions were detected only in PMN and PBMC, with some donor specific variation (Figure 7D and 7E). Unlike total or classical uPAR which were much more highly expressed in peripheral cells, alternative uPAR (E7b) was expressed in lung>PBMC>HASM≈HBEC>THP1≈BEAS2B (Figure 7F). Expression of this variant did not correlate with total uPAR expression. Exon 4+5 deletions were detected in all cells expressing uPAR(E7b) with expression HASM>lung≈HBEC>BEAS2B≈PBMC>THP1 (Figure 7G). Classical uPAR exon 5 and 4+5 deletions and alternative uPAR exon 5 deletion were not detected (data not shown).

**Figure 8:**
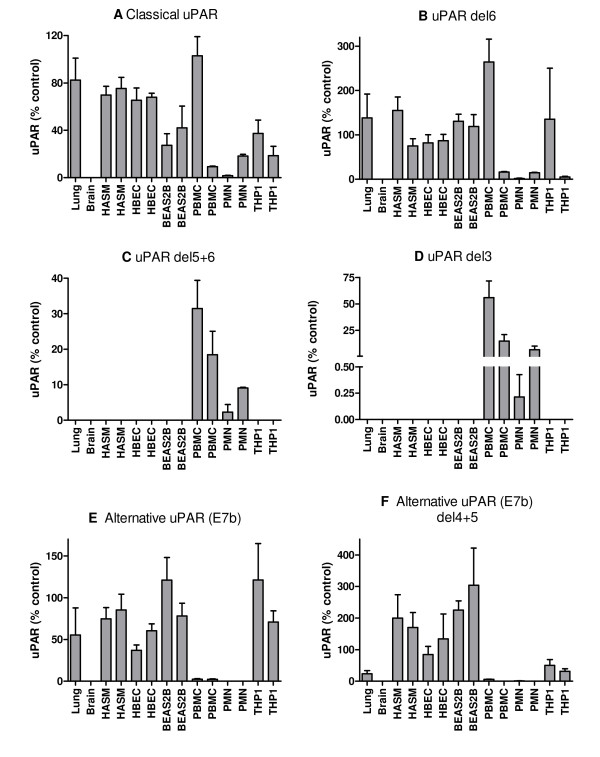
**Expression of different uPAR splice variant mRNAs in different tissues/cell types normalised to total uPAR**. A series of real-time PCR assays was used to measure the expression of different splice variants of uPAR in an extended panel of tissues/cell types (lung, brain, HASM, undifferentiated HBEC, BEAS2B, PMN, PBMC, THP1). Expression of each variant is shown as mean + SEM of three PCR replicates, for two donors or biological replicates as appropriate. Data are shown as 2^-ΔCt ^normalised to total uPAR and relative to a suitable plasmid control (designated 100%). (A) total classical uPAR (exon 7), (B) classical uPAR exon 6 deletion, (C) classical uPAR exons 5+6 deletion, (D) classical uPAR exon 3 deletion, (E) total alternative uPAR (exon7b), (F) alternative uPAR exon 4+5 deletion. Classical uPAR exon 5 and 4+5 deletions and alternative uPAR exon 5 deletion were not detected.

Normalisation of these data for total uPAR expression allowed direct comparison with our previous RACE data which highlighted proportional expression of different splice variants in tissues/cells (Figure 8). The correlation between RACE data (Figure 3) and classical uPAR proportional expression (Figure 8A) was not clear as it may be expected that the proportional expression of this isoform should be elevated in *e.g*. PBMC/PMN vs. lung/HASM/HBEC as shown in the absolute expression data (Figure 7A). However, uPAR(E7b) was proportionally highly expressed in cultured cells (THP1 and BEAS2B) > lung, HASM and HBEC (about 2/3×) >> (1/50×) PBMC (not detected in PMN) (ANOVA not significant) (Figure 8E). These data correspond with RACE data (Figure 3), suggesting lower proportional expression of this variant in primary peripheral cells versus lung cells and highlighted differences between cultured cell lines (BEAS2B, THP1) and the equivalent primary cells *i.e*. HBEC and PBMC. Comparison of the proportional expression of the exon 4+5 deleted uPAR(E7b) form to total uPAR(E7b) expression, suggests lower expression in lung, PBMC and THP1 than HASM, HBEC and BEAS2B (Figure 8F), potentially suggesting that this splicing event occurs preferentially in structural cells. The predicted protein sequences of all confirmed splice variants are shown in Figures 4 and 5.

### Protein analysis confirms the existence of multiple forms of uPAR

**Figure 9:**
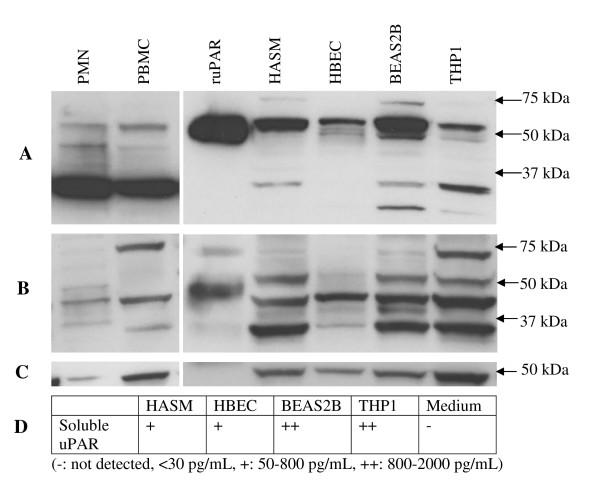
**Expression of uPAR protein in different cell types**. Western blotting of cell lysates was performed for an extended panel of cell types (HASM, undifferentiated HBEC, BEAS2B, THP1, PMN, PBMC) plus recombinant uPAR (ruPAR) using domain I specific (A) and domain II specific (B) antibodies to identify different variants. A β-actin antibody was used as a loading control (C). An ELISA assay, with a sensitivity of 30 pg/mL, was used to detect soluble uPAR in the culture supernatant where appropriate (D).

To determine whether the variation in uPAR isoforms at the mRNA level was reflected at the protein level, total cell lysates were assayed using two uPAR specific antibodies in Western blots. Lysates from two donors or extractions (cell lines) were assayed (except for commercial lysates: PMN and PBMC). Representative results are shown in Figure 9. Western blots performed with either domain 1 or domain 2 specific antibodies detected multiple proteins in all samples (Figure 9A and 9B, summarised in Table 3). Controls using no primary antibody or an isotype control showed no binding to either a THP1 cell lysate or recombinant uPAR (data not shown). Loading was evaluated using β-actin and was equivalent for key comparisons *e.g*. between PBMC and THP1 and between HASM, HBEC and BEAS2B (Figure 9C).

**Table 3:** Identification of proteins in Western blots

	ruPAR	HASM	HBEC	BEAS2B	THP1	PMN	PBMC	Predicted variants
		70		70				dimer/aggregates
	**50–60**	**60**	**60**	**55–60**	**60**	60	60	full length
anti-D1			(55-50)	50	(50)			alternative ex7b
(IIIF10) (kDa)						45	(45)	single exon deletion (*e.g*. exon 3 or 6)
		(37)		(37)	**37**	**37**	**37**	two exon deletions (*e.g*. exon 5+6)
				30	(30)			alternative ex7b, exon 4+5deletion

	75	(75)		(75)	75		**75**	dimer/aggregates
anti-D2	**50–55**	60	(60)	60	60	50		full length
(3932) (kDa)		45	**45**	45	**45**	**45**	**45**	single exon deletion or alternative ex7b
			(40)	40		40		two exon deletions
	(37)	**37**	(37)	**37**	**37**		37	alternative ex7b, exon 4+5deletion or D2/3 fragment

Western blots were performed using two antibodies specific to domains 1 and 2 of the uPAR protein respectively (Figure 9). The main proteins observed in each cell or tissue type with each antibody are summarised. Approximate molecular weights of bands are shown (kDa), with weaker bands shown in brackets and the strongest band in bold. Potential splice variants for each molecular weight are also shown.

Predicted sizes for all variants detected by real-time PCR suggested that multiple variants between 60–100% of the full length classical uPAR should be detected in all cell types (Table 4). It should be noted that additional mRNA species were detected by PCR and RACE which were not analysed by real-time PCR, therefore more species may actually be present in cells. However, the abundance of these transcripts in RACE was low (<5%). The epitope of the D1 antibody is located at amino acids 52–60 (exon 2–3) therefore this antibody will detect all variants except exon 3 deletions and D2/3 proteolysis fragments. The epitope of the D2 antibody is located at amino acids 125–132 (exon 4) therefore this antibody will not detect exon 4 deleted variants or D1 proteolysis fragments. Glycosylation of uPAR occurs at asparginine residues located at amino acids 52 (located in D1, exon3), 162 (D2, exon 5), 172 (D2, exon 5) and 200 (D3, exon 6) and shows site specific heterogeneity, generating multiple different molecular weight forms [25]. This may result in a wide range of proteins on Western blotting.

**Table 4:** Predicted protein sizes and antibody specificity

Variant	Size (aa)*	Predicted MWt (kDa)	Predicted expression (real-time PCR)					
			HASM	HBEC	BEAS2B	PBMC	PMN	THP1
Classical	283	31.5	++	++	+	+++	+++	+
Alternative Exon7	259	28.9	+++	+++	++	+++	-	++

Classical del3	235	26.2	-	-	-	++	++	-
Classical del5+6	189	21.2	-	-	-	++	++	-
Classical del6	234	26.1	++	++	+	+++	+++	+

Alternative del 4+5	160	17.8	+++	+++	++	++	-	+

D1 fragment	83/89	9.8	++	++	++	++	++	++
Classical D2/3	196	21.8	++	++	+	++	++	+
Alternative D2/3	172	19.2	++	++	++	++	-	++

* After removal of signal peptides

Predicted protein translations were performed for variants detected in real-time PCR assays (Figures 4 and 5) and predicted molecular weights (MWt, kDa) obtained. Note that these are potentially about 10 kDa smaller than might be detected at the protein level due to glycosylation. For each variant, relative expression based on real-time PCR results is summarised where +++: highest expression, ++: expression, + low expression and -: not detected. Other variants were detected in RACE experiments, which may result in expression of additional protein variants. Note that the D1 antibody would detect all variants except exon 3 deletions and D2/3 fragments, whilst the D2 antibody would not detect exon 4 deletions or the D1 fragment.

Full-length uPAR protein has a predicted molecular weight of 31 kDa, however differential glycosylation patterns result in a size range of 40–60 kDa. Recombinant uPAR expressed in a mouse melanoma cell line was included and was detected between 45–60 kDa with both antibodies. The D2 specific antibody also detected a higher molecular weight protein (70–75 kDa), possibly representing a dimerisation or aggregation product. A lower molecular weight protein was also observed (30–35 kDa), which probably corresponds to a D2/3 proteolytic fragment.

The D1 antibody detected fewer proteins than the D2 antibody (including for the recombinant protein) (Figure 9A). D1 proteolytic fragments (expected molecular weight 10–20 kDa) were not detected, probably due to their relatively small molecular weight. The highest molecular weight protein seen in all cell types (60 kDa) probably represents full-length classical uPAR as it corresponds most closely to the band observed for recombinant uPAR and by others [26-28]. Proteins are seen around 50 kDa for all except PMN and PBMC cells, suggesting that these may represent the alternative exon 7b form (expected ~90% of the mass of classical uPAR). The slightly smaller fragments detected in PMN and PBMC cells only (designated 45 kDa) may represent single exon (*e.g*. exon 6) deletions of classical uPAR (expected ~80% of the mass of classical uPAR). A protein at 37 kDa is detected in all cell types except HBEC, being particularly strongly expressed in PMN and PBMC. This could represent dual exon deletions (*e.g*. classical uPAR, exon 5+6 deletion), although its pattern of expression does not completely correspond to that seen at the mRNA level. The smallest protein seen (30 kDa) may represent an alternative exon 7b, exon 4+5 deleted form (expected ~55% of the mass of classical uPAR), although this might be expected to be expressed in a greater number of cell types.

The D2 antibody detects three proteins in the recombinant sample, probably reflecting a dimerisation, full-length uPAR and a D2/3 proteolytic fragment (Figure 9B). The large (75 kDa) protein is detected in the majority of cell types, being most strongly expressed in PBMC and THP1 cells. HBEC lysates showed less abundant proteins at lower molecular weights than other structural cell lysates, reflecting the pattern using the D1 antibody, although overall it is difficult to make direct comparisons between the two blots.

The expression of soluble PAR was detected in the supernatants of all cells tested (Figure 9D). Overall, higher levels of soluble uPAR were observed in supernatants from BEAS2B and THP1 cells compared to HASM and HBEC. Interestingly, in the proportional mRNA level analyses, BEAS2B and THP1 were shown to express a greater proportion of the alternative, soluble form (exon 7b) compared to HASM and HBEC (Figure 8E).

### siRNA confirms the specificity of anti-uPAR Western blots

**Figure 10:**
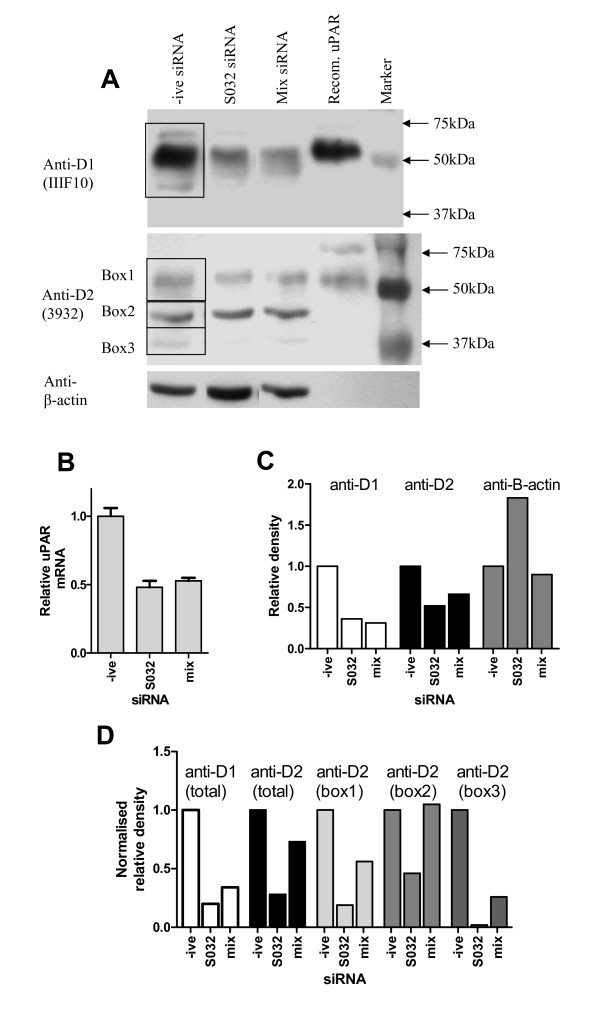
**siRNA knockdown of uPAR in human bronchial epithelial cells**. HBEC were treated with siRNA (negative control, uPAR specific (S032) or pooled siRNA panel (mix)) for 24 hours before RNA and protein extraction. Western blotting was performed using two different anti-uPAR antibodies and an anti-β-actin antibody. Recombinant uPAR (ruPAR) was run as a control (A). Real-time PCR was performed using a total uPAR assay and normalised using HPRT as a housekeeping gene (B). Densitometry was performed on Western blots (regions measured shown boxed) to allow semi-quantitative measurement of knockdown. Data are shown before (C) and after (D) normalisation for β-actin expression. The anti-D2 antibody detected multiple different weight species. Densitometry was performed for individual bands and normalised for β-actin expression (D).

Western blotting using two monoclonal antibodies for uPAR detected multiple bands. To confirm that these bands represent uPAR variants, siRNA was performed in undifferentiated HBEC cells. Cells were treated with siRNA (negative control, uPAR-specific (S032) or a uPAR-specific panel (mix)) for 24 hours before RNA and protein were extracted and analysed by real-time PCR and Western blotting (Figure 10A). Total uPAR mRNA expression was measured by real-time PCR, showing about 50% knockdown with both siRNA strategies (Figure 10B). Total uPAR protein expression detected by the domain 1 (IIIF10) and domain 2 (3932) antibodies is also reduced (Figure 10A) which was confirmed by densitometry analysis, before and after normalisation to β-actin (Figures 10C and 10D respectively). The domain 2 specific antibody detected three main species, which were all shown to be knocked down at the protein level by at least one siRNA approach using densitometry (Figure 10D).

## Discussion

The expression and functional activity of uPAR is of interest in both cancer and respiratory disease. The current study aimed to characterise the key regulatory regions of *UPAR*, identify all common splice variation and quantify expression in the normal lung and in specific lung and peripheral cells at the mRNA and protein levels. We have identified a localised TSS with some cell specificity and confirmed expression of uPAR in lung and peripheral cells. At the mRNA level, multiple uPAR splice variants were identified including alternative E7 (E7b) and deletions of E3, E5+6 and E6, and their patterns of expression in different tissues/cell types characterised. Primary peripheral cells (PMN and PBMC) expressed multiple exon deleted forms of membrane bound uPAR, whilst lung cells including epithelium and airway smooth muscle expressed a greater proportion of an alternative soluble uPAR with and without an exon 4+5 deletion. Protein analyses confirmed expression of multiple differentially expressed forms of uPAR in all cell extracts, and soluble uPAR was detected in the supernatants of cultured HASM, HBEC, THP1 and BEAS2B cells. Specificity of the Western blotting analyses was confirmed by siRNA. Our data provide a novel insight into the molecular mechanisms that potentially regulate uPAR expression and activity in the airways and the periphery which has implications regarding the potential role of uPAR in airway disease biology.

Previous analyses in cell lines suggested the TSS of *UPAR *is 52 bp upstream of the ATG, with a minor start site around -77 bp in U937 (monocytes) and HeLa (epithelial) cells [29]. We have now confirmed and extended these initial analyses using tissues and cells of the respiratory and immune systems; namely lung, HASM, HBEC, PMN and PBMC. In all samples the TSS was located between -42 and -77, however there were clear cell specific locations within this interval *i.e*. -46 to -52 is the dominant TSS in most cells and lung tissue but not in HBEC where the -75 to -78 locus is dominant. The functional significance of the cell specific TSS usage remains to be resolved although it is interesting to note that functional studies using promoter-reporter approaches have suggested that most transcriptional activity is found in the first 220 bp upstream of the ATG in HeLa, HCT116 and RKO (colon cancer) cells [29-31]. Functional studies have implicated AP1 (-122) and SP1-like motifs (spanning -139 to -154) as being important for basal uPAR transcription in colon cancer and HeLa cells [29,30]. We used data-mining to characterise relevant transcription factor binding sites in the 4 kb promoter region identifying a number of different potential pro-inflammatory transcription factor binding sites including AP1, STAT, GATA and CCAAT/EBP sites using two or more databases. Only a single AP1 (-236) site and no SP1 sites were replicated, despite their known importance, indicating that some sites may be missed using our replication strategy, however, the AP1 (-122) and SP1 (-151) sites identified in functional studies were detected by individual databases. uPAR expression has been shown to be regulated by a number of growth factors at the transcriptional level, which include TGFβ1 utilizing the AP1 site at -236 [32]. TGFβ1 is a pro-fibrotic cytokine that has been implicated in airway remodelling in asthma and therefore it is tempting to speculate that TGFβ1 induction of uPAR may be a significant mechanism involved in airway remodelling.

The second objective of the current study was to determine uPAR splice variation in airway and peripheral cells using real-time PCR. Two donors were used for each cell type (but not tissues), to allow for inter-individual differences. We observed expression of the previously identified uPAR E7b soluble splice variant [24], which is predicted to result in the loss of the GPI anchor, in the lung and periphery. Interestingly the primary peripheral cells expressed low levels of this variant (as a fraction of total uPAR) compared to the lung tissue and cells. This suggests that peripheral cells could retain more of their uPAR at the cell membrane (GPI-bound) whilst lung structural cells produce more soluble, secreted uPAR. Binding of uPA to membrane bound uPAR initiates many intracellular signalling pathways, resulting in outcomes including differentiation, proliferation and cell motility [5], whilst soluble uPAR can act as a chemoattractant for hematopoietic cells [33]. It is therefore possible that airway cells might express more soluble uPAR to allow them to attract cells into the airways, whilst the peripheral cells express mainly the surface form of the receptor to allow them to respond to external signals.

In addition to the exon7 variants we also identified a series of internal exon deletion variants expressed within the context of both the membrane bound and soluble receptor. These included exon 3, 4+5, 5+6 and 6 deletions. Exon deleted forms of classical uPAR were found most frequently in the primary peripheral cells, PMN and PBMC. Exon 6 deletion was the most widely expressed. Loss of exon 6 would disrupt the structure of D3, particularly as it includes one of the key cysteines involved in disulphide bonding. This would be expected to lead to reduced uPA binding affinity [19] as two amino acid residues shown to be involved in uPA binding are located in this exon [17]. Expression of an exon 4+5 deleted variant of uPAR has previously been described and was shown to be associated with shorter disease-free survival in breast cancers using a real-time PCR strategy [3], however the assay used did not distinguish between exon 7 variants. In the present study, we did not detect this deletion in combination with classical exon 7 using real-time PCR (although a single clone was obtained from PBMC by PCR), whereas in combination with alternative exon 7b this variant was detected in most tissues/cell types, notably the structural cells HASM, HBEC and BEAS2B. Based on the known structure of uPAR, this variant might express a soluble form of the receptor lacking the key chemotactic sequence [22] and D2, which includes integrin binding domains [20]. Some uPA binding ability conferred by D1 may be retained [19] although many uPA binding residues will be lost [17] and the structure of the protein [18] will be compromised. It is possible that this form might act as a dominant-negative inhibitor of uPAR action, by sequestering uPA and preventing its binding to full length membrane-bound or soluble forms of the receptor.

Repeated epithelial wounding/repair leading to airway remodelling has been implicated in asthma pathogenesis [34]. Epithelial cells have the capacity to repair by cell spreading, migration and proliferation, all integrin-dependent processes. Integrins have therefore been suggested to play a prominent role in wound repair in asthma and the expression of α3β1 (laminin receptor) and α5β1 (fibronectin receptor) integrins have been shown to be upregulated at epithelial wound edges [35,36]. uPAR interacts with integrins (mainly α3β1 and α5β1) and has been shown to influence cell adhesion and migration on ECM proteins [37,38]. Therefore the identification of splice variants that lose this ability to bind integrins (*i.e*. exon 5 deletions) may be of significance in normal and disease mechanisms.

At the protein level, multiple forms of uPAR were detected in cell lysates. Overall, our data suggest that multiple forms of uPAR are expressed in the panel of cells studied and there is some evidence of differential expression. This corresponds to the findings of Brooks *et al*. [27], who showed predominantly lower molecular weight forms of uPAR to be expressed in peripheral neutrophils, whilst higher molecular weight forms were more highly expressed in lung eosinophils. These data are also in keeping with our mRNA analyses which suggested a higher proportion of exon deletion variants are expressed in the periphery, potentially resulting in lower molecular weight proteins.

Although the majority of high abundance predicted uPAR variants might be expected to be detected using either the D1 or D2 antibody, the patterns of expression observed were markedly different. This could reflect differing affinities of the two antibodies for different variants. It should be noted that the epitope for the D1 antibody (amino acids 52–60) corresponds to one of the potential glycosylation sites for the protein (Asn^52^) [25]. The presence of a large carbohydrate side-chain may prevent binding of the antibody, resulting in detection of fewer forms by this antibody as we have observed. Additionally, the D1 antibody will not detect D2/3 proteolytic fragments, explaining the presence of fewer low molecular weight proteins detected using this antibody. An attempt has been made to mirror patterns of protein expression as determined by Western blot to real-time PCR results, with some success for the D1 antibody results *e.g*. the presence of a 50 kDa protein potentially representing the alternative exon7b observed in all cell types except PMN and PBMC, as well as a 45 kDa form seen only in PBMC and PMN which may represent single exon deletions of classical uPAR. Concordance between the two assays may be affected by differing efficiencies of the real-time PCR assays or antibody specificity. However, it will also reflect genuine biological differences, as post-translational control mechanisms including glycosylation and proteolytic cleavage play a key role in uPAR protein maturation [15,25]. Our data reflect this complex regulatory pathway.

All cultured cells tested showed expression of soluble uPAR. This assay does not distinguish between classical uPAR released from its GPI anchor, proteolytic fragments and alternatively spliced (exon 7b) soluble uPAR. However, all of these cell lines expressed relatively high proportions of alternative uPAR(E7b) mRNA and the level of mRNA correlates with soluble uPAR protein expression *e.g*. BEAS2B and THP1 had elevated alternative uPAR(E7b) mRNA and soluble uPAR protein. Therefore, the elevated levels of uPAR in the sputum of asthma and COPD subjects may reflect expression of soluble uPAR from both epithelial and smooth muscle cells.

## Conclusion

We have identified the key promoter region of *UPAR *in the airway and highlighted potential regulatory transcription factor binding sites. We have identified multiple splice variants with potentially different functional activity including soluble expression and/or the loss of key binding domains, such as those involved in ligand binding or integrin interactions. The expression patterns of these splice variants in airway and peripheral cells provide an insight into the molecular mechanisms regulating uPAR expression and activity. These data highlight the importance of uPAR splice variants, which should be taken into consideration in future work to determine the role of uPAR in respiratory disease.

## Methods

### Cell culture, preparation of total RNA and cell protein lysates and siRNA knock-down

Primary human airway smooth muscle (HASM) cells were isolated and prepared as described previously [39] and cultured in DMEM + 10% foetal calf serum (FCS). Airway smooth muscle cells from two individuals were used. BEAS-2B (airway epithelial cells) were cultured in DMEM + 10% FCS, whilst THP1 (monocyte) cells (ATCC) were cultured in modified RPMI 1640 medium + 10% FCS + 0.05 mM β-mercaptoethanol. Human bronchial epithelial cells (HBEC) from two donors were obtained from Lonza (Wokingham, UK) and cultured in bronchial epithelial growth medium (BEGM). Cells were differentiated at an air-liquid interface using bronchial epithelial differentiation medium (BEDM) as described previously [40].

For downstream analysis, cells were lysed and RNA extracted using silica columns (RNeasy mini kit, Qiagen, Crawley, UK). For protein analysis, cell lysates were prepared using cytobuster buffer (Novagen, Merck, Nottingham, UK) with benzonase (Merck) and complete protease inhibitor (Roche, Welwyn Garden City, UK), as directed by the manufacturer. Ethical approval for the use of primary cells was obtained from the Nottingham University Hospitals local ethical committee.

uPAR was targeted for knockdown in undifferentiated HBEC cells using siRNA specific to exon 1 (S032) or a panel of four siRNAs specific to exons 1–2, 7 and 7b (mix). Silencer select negative control #1 was used as a control. siRNA sequences available on request. Silencer select siRNAs were obtained from Applied Biosystems (Warrington, UK). Cells were plated in 6-well plates at 100,000 cells per well and transfected after 48 hours with 10 nM total siRNA using 5 μl Liopfectamine 2000 as directed by the manufacturer (Invitrogen, Paisley, UK).

### Peripheral cell preparation

Whole blood was collected from healthy volunteers (45 ml). Red blood cells were removed using dextran sedimentation, before separating the neutrophils (PMN) and mononuclear cells (PBMC) by centrifugation in a discontinuous percoll gradient. Slides were prepared by cytospin for characterisation of cell populations. PMN were >95% pure (some eosinophils and basophils), whilst PBMC consisted mostly of lymphocytes (44%) and monocytes (39%) with the remainder eosinophils and neutrophils as defined by morphology. Ethical approval was obtained from the Nottingham University Hospitals local ethical committee. RNA was prepared as described previously. RNA and lysates from PMN and PBMC were also obtained commercially (3 H Biomedical, Uppsala, Sweden).

### Rapid amplification of cDNA ends (RACE)

RACE-ready cDNA was synthesised from total RNA (1 μg) (PMN, PBMC, HASM, differentiated and undifferentiated HBEC and commercially obtained lung (Ambion, Applied Biosystems)) using the GeneRacer kit as directed (Invitrogen). 5' and 3' RACE used GeneRacer primers and uPAR exon 4 specific nested primers for amplification (Table 2). Full-length uPAR PCR products generated using primers specific to exon 1 and 7 or 7b were also cloned to further clarify the exon structure of the gene. Both RACE products and PCR products were sequenced using BigDye 3.1 (Applied Biosystems) in conjunction with an ABI 310 DNA sequencer. Sequences were aligned to the human database using BLAST but also compared to known uPAR transcripts and each other using EMBL-EBI's multiple alignment tool, ClustalW . Alignments were made against reference sequences NM_002659.2 (classical E7) and NM_001005376 (alternative E7b).

### Real-time PCR

mRNA levels of total uPAR and splice variants were quantified using a series of real-time PCR assays. Amplicons spanning exons 1–2, 1–7 and 1–7b were used in combination with a series of splice-variant specific FAM/TAMRA labelled TaqMan probes to define up to 16 different variants (Table 2, Figure 6). For each splice variant, PCR products were cloned and sequenced to generate a positive control and standard. cDNA was synthesised using Superscript II (Invitrogen) and random hexamer primers as per instructions. RNA (0.7 μg) was used in a 20 μl reverse transcription reaction. Real-time PCR was performed using TaqMan gene expression master mix (Applied Biosystems) and 18s ribosomal RNA or HPRT1 endogenous control (Applied Biosystems). Data was corrected using the housekeeper (18s or HPRT1) and the 2^-ΔCt ^method and normalised to a variant specific positive control plasmid. Alternative splice variants were also normalised to total uPAR to identify proportional expression and allow comparison to RACE data.

### Western blotting

Lysates of cultured cells and commercially obtained PMN and PBMC protein lysates were separated by 10% SDS-PAGE under reducing conditions. Proteins were transferred to a PVDF membrane and probed using two anti-uPAR antibodies; uPAR D1 monoclonal: IIIF10 (Santa Cruz Biotechnology, Heidelberg, Germany) 0.25 μg/ml (1:400) and uPAR D2 monoclonal: 3932 (American Diagnostica, Axis-Shield UK, Kimbolton, UK) 2.5 μg/ml (1:200) with an appropriate HRP-conjugated secondary antibody (R&D systems, Abingdon, UK) (1:1000). Binding was visualised by enhanced chemiluminescence (ECL, Amersham Biosciences, GE healthcare, Little Chalfont, UK). Membranes were stripped using Restore Western blot stripping buffer (Thermo Fisher Scientific, Perbio Science UK, Cramlington, UK) and re-probed for β-actin expression (ab8227, Abcam, Cambridge, UK, 1:5000) as a loading control. Antibody dilutions were based on manufacturers' recommendations. Densitometry of protein bands was completed using ImageJ 1.41 .

### Enzyme-linked immunosorbent assay (ELISA)

The uPAR Duoset ELISA kit (R&D systems) was used to quantify soluble uPAR in cell culture supernatants, following the manufacturer's recommendations. uPAR was quantified using tetramethylbenzidine (R&D systems) and a MultiscanEX plate reader (Thermo Fisher Scientific) at 450 nm with 570 nm background subtraction. The detection limit of the assay was 30 pg/mL sample.

### *In silico *promoter analysis

Transcription factors relevant to respiratory disease and asthma were identified by reference to the literature (AP1, NFKB, NF-AT, CREB, STAT, C/EBP, GR, RXR, SRC-1, TIF-2, p/CAF, CBP, GATA)[41,42]. A 4000 bp fragment upstream of the transcriptional start site was analysed for potential transcription factor binding sites using four different algorithms available online, as described previously [39]. Results of these searches were compared and those binding sites identified in two or more different searches were included in a final summary.

### Statistical analysis

Differences between RACE results were evaluated using contingency tables (Chi-square test). Differences in expression in real-time PCR experiments were evaluated using ANOVA and Tukey's *post hoc *test. Data were analysed using Prism v.5.01 (GraphPad software, La Jolla, CA). P < 0.05 was considered significant.

## Abbreviations

COPD: chronic obstructive pulmonary disease; D1–3: domain 1–3 (of uPAR protein); ECM: extracellular matrix; ELISA: enzyme-linked immunosorbent assay; GPI: glycosylphosphatidylinositol; HASM: human airway smooth muscle cell; HBEC: human bronchial epithelial cell; HPRT1: hypoxanthine guanine phosphoribosyltransferase 1; PBMC: peripheral blood mononuclear cell; PCR: polymerase chain reaction; PMN: polymorphonuclear cell (neutrophil); RACE: rapid amplification of cDNA ends; SEM: standard error of the mean; TSS: transcriptional start site; uPA: urokinase plasminogen activator (PLAU); uPAR: urokinase plasminogen activator receptor (PLAUR); UTR: untranslated region.

## Authors' contributions

CS participated in the design of the study, performed the benchwork and statistical analyses and drafted the manuscript. IS conceived the study, participated in its design and coordination and helped to draft the manuscript. All authors read and approved the final manuscript.
